# Effect and safety of a physical exercise-based intervention on body composition and cardiometabolic health of adolescents with severe obesity. Secondary analysis from the PAC-MAnO trial

**DOI:** 10.1016/j.obpill.2025.100190

**Published:** 2025-06-26

**Authors:** Antonio Videira-Silva, Fábio de Freitas, Luis B. Sardinha, Helena Fonseca

**Affiliations:** a WHO Collaborating Centre for Adolescent Medicine and Training, Clínica Universitária de Pediatria, Faculdade de Medicina, Universidade de Lisboa, Lisbon, Portugal; b CIDEFES (Centro de Investigação em Desporto, Educação Física, Exercício e Saúde), Universidade Lusófona, Lisbon, Portugal; c CIFI2D (Research Centre in Physical Activity, Health and Leisure), Universidade do Porto, Porto, Portugal; d Unicamp School of Medical Sciences, Department of Pediatrics, Child and Adolescent Health Graduate Program, Campinas, São Paulo, Brazil; e Exercise and Health Laboratory, CIPER, Faculty of Human Kinetics, Universidade de Lisboa, Lisbon, Portugal; f Pediatric Obesity Clinic, Department of Pediatrics, Hospital de Santa Maria, Lisbon, Portugal

**Keywords:** Adolescents, Severe obesity, Pediatric obesity, Weight loss, Physical activity, Exercise

## Abstract

**Background:**

Severe obesity (SOb) treatment in adolescents has been focused on surgery and pharmacological treatment, in part due to difficulty in implementing lifestyle behavioral changes, such as physical exercise. However, the potential long-term consequences and costs of these options prompt new effective and sustainable treatment options. This study aimed to investigate the effect and safety of a 6-month high-intensity physical exercise-based intervention on body composition and cardiometabolic health in adolescents with SOb (BMI z-score ≥3), compared to those without SOb and SOb controls (without intervention).

**Methods:**

Data from 46 adolescents (50 % girls), including 20 with SOb (43.5 %), exposed to two exercise sessions/week for 6 months, and 16 controls were analyzed.

**Results:**

Adolescents with SOb showed a higher decrease and increase in central fat mass (*β=*-2.3, 95 %CI: 4.8, 0.1) and fat-and-bone-free mass (*β* = 1.70, 95 %CI: 3.25, 0.15) and a higher rate of insulin resistance normalization (58.3 vs. 12.5 %) after the intervention, compared to those without SOb, with no differences in adverse effects. Adolescents with SOb exposed to intervention also showed improvements in BMI, compared to an impairment in controls (95 %CI: 2.64, 0.16).

**Conclusion:**

High-intensity physical exercise-based interventions should be considered as an obesity treatment option in adolescents with SOb. Higher exercise volume and/or frequency may further improve health-related outcomes and should become a first-line strategy alongside other lifestyle changes.

**Trail registration:**

Clinicaltrials.gov NCT02941770.

## Highlights

• High-intensity exercise program improved body composition and insulin resistance in adolescents with severe obesity (SOb).
• Adolescents with SOb responded equally or more favorably than no-SOb, without serious adverse effects.
• Consistent attendance (≥80 % of sessions) was associated with greater improvements, emphasizing the importance of adherence.

## Graphical abstract

**Figure:**
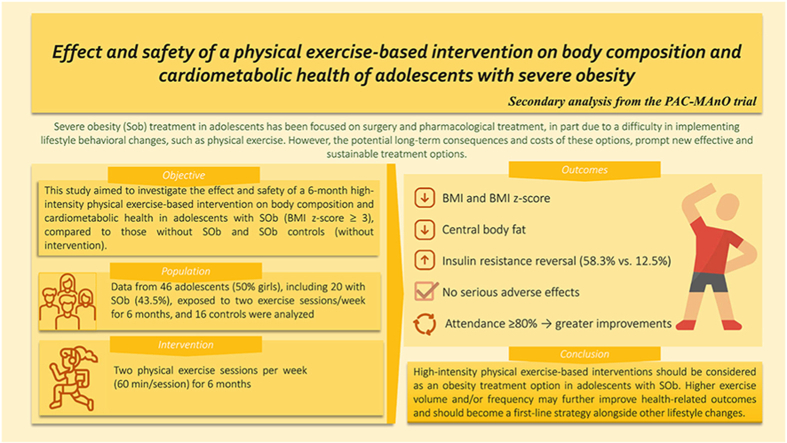

## 1 Introduction

Pediatric obesity is still a public health concern worldwide [1], being associated with several adverse health conditions [2] and increased morbidity and mortality in adulthood [3].

Although, in the last decade, the prevalence of obesity has achieved a plateau in most of the developed countries, the incidence of severe obesity [SOb, i.e., a body mass index (BMI) z-score ≥3 [4] has scaled [5,6], hastening the development of comorbidities, impairing the future of children and adolescents [7], and increasing health care seeking and costs [8].

Current management of SOb in adolescents is limited in effectiveness and often inaccessible, not only due to high costs but also because of regional variations in medication approval and availability [9]. For instance, bariatric surgery, although effective, has a high cost for families and governments, being potentially associated with intraoperative, short, and long-term complications [10]. On the other hand, pharmacological treatment (e.g., Glucagon-like peptide-1 receptor agonists, GLP1-RA) are associated with more modest results and side effects, mainly affecting the gastrointestinal tract [11]. In addition, weight-loss maintenance under pharmacological treatment depends on treatment consistency, which has been reported as low in adults [12], and on lifestyle changes, including improvements in nutritional and physical activity (PA) behaviors, that are crucial to the success of both therapeutic strategies [10,11].

Vigorous PA and high-intensity physical exercise have shown potential benefits in obesity treatment among adolescents, despite individual variability observed in response to physical exercise [13]. Yet, among adolescents with SOb, decreased perceived self-efficacy and enjoyment in PA [14], especially in those with higher intensities, may lead to low adherence to physical exercise programs, compromising the possible results in this specific population. Moreover, it is still a matter of concern whether high-intensity physical exercise is safe for those with SOb or even if it has a similar effect on overall health compared to those without SOb.

Although SOb management in adolescents has been focused on surgery and pharmaceutical treatment, the potential long-term consequences and high healthcare costs associated with those strategies prompt “new” effective and sustainable treatment options targeting this sub-group of adolescents who are at high risk of metabolic and cardiovascular disease development. Thus, the main aim of this study was to investigate the effect and safety of a 6-month high-intensity physical exercise-based intervention on body composition and cardiometabolic health in adolescents with SOb, compared to those without SOb.

## 2 Methods

### 2.1 Study design

Study design and outcomes are reported according to CONSORT-Outcomes 2022 Extension [15].

#### 2.1.1 Trial design

The present exploratory study used data from the PAC-MAnO trial (Clinicaltrials.gov NCT02941770) [16]. Briefly, PAC-MAnO consisted of a 3-arm controlled trial (6-month intervention plus 6-month follow-up) testing the efficacy of a PA consultation [including weekly supervised physical exercise sessions –Experimental Group (EG) II, or not including the physical exercise sessions – EG I] compared to standard care (control group - CG), as part of a multidisciplinary program for the management of adolescent obesity [16].

### 2.2 Participants

A total of 165 adolescents aged 14.6 (±1.8) years, with a BMI z-score of 3.0 (±0.8), attending for the first time a Pediatric Obesity Clinic at a European Public Central Hospital, were enrolled in the original trial (CG, *n* = 55; EGI *n* = 55; EGII *n* = 55) [17]. This study used data from 46 (83.6 %) EGII participants who completed the 6-month physical exercise intervention. No differences at baseline were found in BMI or BMI z-score between the participants included in the analysis (*n* = 46) and those who dropped out of the study (n = 9), neither between groups (CG/EGI/EGII). Additional analyses were performed, including 6-month data from 16 participants with SOb allocated to the CG.

### 2.3 Exercise sessions

EGII participants were invited to attend two physical exercise sessions per week (60 min/session) for 6 months (phase I), facilitated by one exercise physiologist.

Exercise sessions included 13–15 min of warm-up (30–50 % heart rate - HR reserve); 35–37 min of resistance (using body weight) and aerobic training (60–100 % HR reserve); and 10 min of cool-down (30–50 % HR reserve) [18].

Resistance exercises included modified push-ups (against the wall, bench or on the floor with the knees), elevations (not supporting total body mass), front raise, bicep curl, triceps dip (using a step platform), and abdominal crunch for the upper body; and squats (isotonic or isometric), lunges (stationary or with movement – restricted range; one leg or alternated), leg extension (on the floor in the supine position), and back bridge (with one or both legs) for the lower body [18].

### 2.4 Outcomes

The effect of exercise was based on anthropometrics, body composition, and cardiometabolic changes, using the BMI z-score as the primary outcome, and a difference of −0.15 in this variable was clinically significant [19].

Safety was based on the number of adverse events observed/reported.

Sex, Ethnicity, pubertal status, daily PA, and Total energy intake were assessed and analyzed as covariates.

### 2.5 Measurements and instruments

Height was assessed with a height stadiometer (SECA 217, Hamburg, Germany) in the Frankfurt plan, without shoes, after an expiratory phase, and registered to the nearest 0.1 cm.

Body weight was measured with a bioelectrical impedance scale (InBody 230, Seoul, Korea) to the nearest 0.1 kg, with the subjects wearing as few clothes as possible and without shoes or socks.

BMI was calculated as body weight in kilograms divided by the square of height in meters [BMI = weight (kg)/height2 (m)]. BMI z-score was further calculated based on World Health Organization (WHO) data using the WHO AnthroPlus calculator (version 1.0.4).

Waist circumference (WC) was measured with an anthropometric measuring tape (SECA 203, Hamburg, Germany), with the subject standing 1 cm above the iliac crest at the end of a regular expiration. Waist-to-Height ratio (WHtR) was additionally calculated (WHtR= WC/Height).

Pubertal status was objectively assessed by a pediatrician and categorized according to Tanner stages [20].

Body composition was assessed by both bioelectrical impedance (InBody 230, Seoul, Korea) and dual-energy x-ray absorptiometry (DXA) (Explorer W, Hologic; Waltham, MA, USA). DXA data were used to test differences in body composition between EGII participants with and without SOb. Body composition was analyzed using equipment software (QDR 12.4, Waltham, MA, USA). DXA exam was performed following the National Health and Nutrition Examination Survey (NHANES) protocol. Total body fat mass (TBFM), trunk fat mass (Trunk FM), and fat-and-bone-free mass (FBFM) were considered variables of interest. Relative body fat mass (BFM) and muscle mass (MM) were calculated as TBFM and FBFM divided by body weight, respectively, and expressed in percentage (%). Central fat mass (Central FM) was additionally calculated as Trunk FM divided by TBFM and expressed in percentage (%). Bioelectrical impedance data were used only to test differences between participants with SOb from EGII and CG due to the significant missing DXA data in CG [17].

Resting systolic blood pressure (SBP) and diastolic blood pressure (DBP) were measured with a digital sphygmomanometer (CAS 9302S, CAS Medical Systems, Branford, USA) on the right arm with an appropriately sized cuff after 5 min of rest in the seating position. The measurement was performed at least three times, and the average of the three measurements (with differences ≤5 mmHg) was recorded.

Biochemical analyses were performed in the clinical pathology laboratory at the hospital. Blood samples were collected after overnight fasting (12 h) in the presence of one of the parents/caregivers and after a local application of a topical anesthesia patch (EMLA). Blood glucose levels were determined using the hexokinase method, and insulin was assessed using a chemiluminescence immunoassay technique. Insulin resistance (IR) was derived from the homeostasis model assessment (HOMA) method. Total cholesterol (TC), triglycerides (TG), and high-density lipoprotein cholesterol (HDL-C) were determined using enzymatic, GPO-trinder, and direct methods, respectively. Low-density lipoprotein cholesterol (LDL-C) was calculated based on total cholesterol and HDL-C levels. Alanine aminotransferase (ALT) levels were assessed with a modified IFCC method. C-reactive protein (CRP) was determined using a turbidimetric immunoassay (Siemens, ADVIA 2400, Newark, DE, USA). The presence of cardiometabolic comorbidities was determined based on the cutoff values previously published and presented in Supplemental Table 1 [21].

Cardiorespiratory fitness (CRF), i.e., oxygen uptake during peak exercise (VO_2_ peak), was directly determined with a gas analyzer (K4 b2, Cosmed, Rome, Italy) during a submaximal exercise test using a cycle ergometer (electronically braked cycle ergometer, Monark 839 Ergomedic, Monark, Vansbro, Sweden). Initial workload and increments were 40 or 50 W for girls and boys. Heart rate was registered continuously (Polar Vantage NV, Polar Electro Oy, Kempele, Finland). Criteria to stop the test included a heart rate ≥85 % to the theoretical maximal heart rate, failure to maintain a frequency of at least 30 revolutions/min, and a subjective judgment by the observer that the adolescent was exhausted [22]. VO_2_ peak (ml/min) was adjusted for body weight (ml/kg/min) and used in the analyses.

PA was assessed with accelerometers (ACTIGRAPH GT3X, Pensacola, Florida, USA) programmed to use a 5-s cycle during at least one weekend day and two weekdays. Days with more than 480 min (8h) registered were included in the analysis. Activities between 0 and 149 counts/minute were considered as stationary time (i.e., time as sedentary behavior collected from an accelerometer that does not measure posture or context) [23]; between 150 and 499, light PA (LPA); between 500 and 3999, moderate PA (MPA); and more than 4000 counts/minute vigorous PA (VPA) [24]. The analysis calculated and used the daily average of stationary time, LPA, MPA, VPA, and moderate-vigorous (MVPA ​= ​MPA ​+ ​VPA).

Total energy intake (TEI) was estimated through three-day food records (2 weekdays and 1 weekend day) using semi-quantitative scaling (e.g., number of spoons or scoops), according to the 4th edition of *Weight and Food portions*. Three trained nutritionists independently analyzed each food record. Food was converted into nutrients with nutritional information from the Portuguese Food Composition Table. Final data were included in the analyses after a group discussion [17].

### 2.6 Statistical analysis

Data were analyzed using the IBM SPSS statistics (version 28.0, IBM, New York, USA). Descriptive characteristics, i.e., mean ± SD and median (interquartile range, IR), were calculated for normally distributed and skewed outcomes, respectively. Baseline differences between participants with and without SOb were analyzed with independent sample *t*-tests/Mann-Whitney U tests and Chi-squared for continuous and categorical variables, respectively. Generalized Estimating Equations (adjusted for age, sex, Tanner stage, and type of exercise, i.e., High-intensity interval training/Traditional training) were used to analyze over time changes, as well as time-by-group (SOb/non-SOb) and time-by-attendance interactions. An attendance (i.e., the number of sessions attended by each participant) ≥ 80 % of the scheduled exercise sessions was considered optimal.

Additional analyses investigating baseline and 6-month differences between participants with SOb from EGII (exposed to exercised-based intervention) and CG were performed using Independent sample *t*-test and Mann-Whitney *U* test for normally distributed and skewed outcomes, respectively, as well as Chi-squared (for categorical variables).

A *p*-value of ≤ 0.05 was considered statistically significant.

## 3 Results

Participants’ characteristics are shown in Table 1. Of the 46 EGII participants included in the analysis (50 % girls; mean age of 14.3 ± 1.7 years; median BMI z-score of 2.96), 20 (43.5 %) had SOb.

**Table 1:** Participants’ baseline characteristics.

*Outcome*		no-SOb (*n* = 26)	SOb (*n* = 20)		Total (*n* = 46)
		**Mean ± SD**		***p***	**Mean ± SD**
Age (years)		14.4 ± 1.3	14.2 ± 2.1	0.661^a^	14.3 ± 1.7
Weight (kg)		81.6 ± 11.7	104.8 ± 17.1	**<**0.**001** a	91.7 ± 18.3
Height (cm)		162.4 ± 7.7	166.0 ± 9.5	0.158 a	164.0 ± 8.6
BMI (kg/m^2^)		30.39 (5.43)	37.32 (3.50)	**<**0.**001 ^b^**	33.83 (7.47)
BMI z-score		2.61 (0.44)	3.50 (0.55)	**<**0.**001 ^b^**	2.96 (0.91)
WC (cm)		98.4 ± 6.1	117.7 ± 9.9	**<**0.**001** a	106.8 ± 12.5
WHtR		0.61 ± 0.03	0.71 ± 0.06	**<**0.**001** a	0.65 ± 0.07
BFM (%)		42.8 ± 5.2	47.0 ± 4.8	0.**007**	44.6 ± 5.4
MM (%)		31.5 ± 3.1	29.7 ± 3.0	0.057	30.7 ± 3.2
BMC (kg)		1.90 ± 0.35	2.27 ± 0.57	0.**019** a	2.07 ± 0.49
BMD (g/m^2^)		1.01 ± 0.09	1.08 ± 0.14	0.078 a	1.03 ± 0.12
BMD z-score		−0.16 ± 1.11	0.27 ± 1.25	0.305 a	0.02 ± 1.17
		***n* (%)**		***p***	***n* (%)**
Ethnicity (Caucasian)		25 (96.2)	13 (65.0)	0.**021 ^c^**	66 (93.0)
Sex (Girls)		17 (65.4)	6 (30.0)	0.**017 ^c^**	23 (50.0)
Tanner stage	II	4 (15.4)	3 (15.0)	0.289^c^	7 (15.2)
	III	5 (19.2)	1 (5.0)		6 (13.0)
	IV	3 (11.5)	6 (30.0)		9 (19.6)
	V	14 (53.8)	10 (50.0)		24 (52.2)
High BP a		5 (19.2)	11 (55.0)	0.**012 ^c^**	16 (34.8)
Hyperglycemia a		0 (0.0)	0 (0.0)	–	0 (0.0)
IR a		8 (30.8)	12 (60.0)	0.**047 ^c^**	20 (43.5)
Dyslipidemia a		10 (38.5)	10 (50.0)	0.434^c^	20 (43.5)
High CRP a		5 (19.2)	6 (30.0)	0.396^c^	11 (23.9)
High ALT a		3 (11.5)	5 (25.0)	0.232^c^	8 (17.4)
Metabolic Syndrome a		3 (11.5)	3 (15.0)	0.730^c^	6 (13.0)

ALT, alanine aminotransferase; BFM, body fat mass; BMC, bone mineral content; BMD, bone mineral density; BMI, body mass index; BP, blood pressure; CRP, C-reactive protein; HipC, hip circumference; IR, insulin resistance; SOb, severe obesity (BMI z-score ≥3); WC, waist circumference; WHtR, waist-height ratio.

^a, b, c^ Between-group differences analyzed with Independent-sample *t*-test, Mann-Whitney *U* test, and Chi-squared, respectively.

a The cutoff values for metabolic variables are presented in Supplemental Table 1.

At baseline, participants with SOb showed higher weight, BMI, BMI z-score, WC, WHtR, Hip circumference, TBFM, BFM, Trunk FM, Central FM, FBFM, and BMC (*p* < 0.05), compared to those with no-SOb. In addition, a lower percentage of Caucasian participants and girls, as well as a higher number of participants with high blood pressure (HBP) and insulin resistance (IR) were noted in participants with SOb (*p* < 0.05).

No difference was found in attendance between adolescents with and without SOb (participation in ≥80 % of the scheduled exercise sessions).

### 3.1 Within and between-group changes in anthropometrics and body composition

Overall, a time effect was observed in all anthropometric and body composition variables, except in BMD z-score. Improvements in BMI, BMI z-, WHtR, TBFM, BFM, Trunk FM, Central FM, FBFM, MM, BMC, and BMD were found among the included participants (*p* < 0.05). An additional significant increase in weight was found, with participants showing, in general, an increase in weight (*p* < 0.05) (Table 2).

**Table 2:** Within and between-group differences in anthropometrics and body composition, at baseline and over time, in adolescents with/without severe obesity.

	no-SOb (*n* = 26)		SOb (*n* = 20)		Time	Time∗Group	Time∗Attendance
*Variable*	*Baseline*	6 months	*Baseline*	6 months	*β* (95 % CI)	*β* (95 % CI)	*β* (95 % CI)
Weight (kg)	81.6 ​± ​11.7 ∗	82.1 ± 12.6	104.8 ​± ​17.1 ∗	106.1 ± 17.6	**2.6 (0.2, 4.7)**	0.7 (−2.9, 4.3)	**−6.3 (-9.0, -3.7)**
BMI (kg/m^2^)	30.84 ​± ​3.01 ∗	30.59 ± 3.23	37.89 ​± ​4.24 ∗	37.68 ± 4.97	**−0.05 (-0.07, -0.03)**	−0.04 (−1.20, 1.3)	**−2.21 (-3.03, -1.38)**
BMI z-score	2.56 ​± ​0.36 ∗	2.46 ± 0.44	3.72 ​± ​0.57 ∗	3.62 ± 0.70	**−0.10 (-0.13, -0.06)**	−0.02 (−0.18, 0.15)	**−0.28 (-0.40, -0.16)**
WHtR	0.61 ​± ​0.03 ∗	0.60 ± 0.04	0.71 ​± ​0.06 ∗	0.70 ± 0.07	**−0.03 (-0.05, -0.02)**	−0.01 (−0.03, 0.01)	**−0.04 (-0.05, -0.02)**
TBFM (kg)	33.27 ​± ​7.35 ∗	33.07 ± 8.08	43.81 ​± ​8.07 ∗	42.5 ± 8.74	**−3.27 (-5.31, -1.24)**	0.40 (−3.00, 3.79)	**−5.39 (-7.32, -3.46)**
BFM (%)	42.8 ​± ​5.2 ∗	42.0 ± 5.4	47.0 ​± ​4.8 ∗	45.0 ± 6.3	**−3.2 (-4.8, -1.6)**	−0.8 (−2.4, 0.8)	**−1.6 (-4.0, -1.2)**
Trunk FM (kg)	15.10 ​± ​3.55 ∗	14.24 ± 3.71	21.61 ​± ​4.97 ∗	18.79 ± 5.37	**−3.70 (-4.69, -2.71)**	−1.09 (−2.90, 0.72)	**−2.71 (-3.87, -1.54)**
Central FM (%/TBFM)	45.4 ​± ​4.8 ∗	43.1 ± 4.9	49.1 ​± ​5.5 ∗	43.8 ± 6.4	**−5.6 (-7.6, -3.5)**	**−2.3 (-4.8, 0.1)**	−1.2 (−3.5, 1.1)
FBFM (kg)	44.19 ​± ​5.90 ∗	45.15 ± 6.38	54.99 ​± ​11.26 ∗	57.70 ± 10.40	**2.55 (1.08, 4.02)**	**1.70 (3.25, 0.15)**	−0.26 (−1.86, 1.35)
MM (%)	31.5 ± 3.1	32.1 ± 3.2	29.7 ± 3.0	31.0 ± 3.9	**1.9 (0.9, 2.9)**	0.5 (−0.4, 1.4)	**1.4 (0.5, 2.2)**
BMC (kg)	1.90 ​± ​0.35 ∗	1.97 ± 0.36	2.27 ​± ​0.57 ∗	2.29 ± 0.57	**0.12 (-0.04, 0.20)**	0.03 (−0.05, 0.11)	−0.02 (−0.09, 0.06)
BMD (g/m^2^)	1.01 ± 0.09	1.03 ± 0.10	1.08 ± 0.14	1.11 ± 0.16	**0.12 (0.03, 0.22)**	0.01 (−0.01, 0.04)	0.00 (−0.02, 0.03)
BMD z-score	−0.16 ± 1.11	−0.02 ± 1.19	0.27 ± 1.25	0.66 ± 0.88	0.51 (−0.10, 1.12)	0.26 (−0.24, 0.75)	0.18 (−0.24, 0.59)

BFM, body fat mass; BMC, bone mineral content; BMD, bone mineral density; BMI, body mass index; FBFM, fat- and bone-free mass; FM, fat mass; MM, muscle mass; SOb, severe obesity (BMI z-score ≥3); TBFM, total body fat mass; WHtR, waist-height ratio.

∗ Statistically different at baseline (*p* ≤ 0.05).

Bold β s indicates a significant (*p* ≤ 0.05) time, time-by-group (SOb), and time-by-attendance (≥80 % of all exercise sessions), respectively. Standardized β s adjusted for age, sex, Tanner stage and type of exercise (High-intensity interval training/Traditional training).

**Fig. 1:**
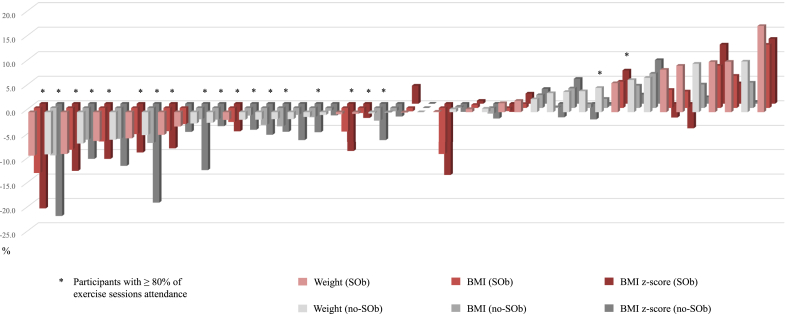
Relative changes (% of baseline values) in weight, BMI, and BMI z-score in participants with and without severe obesity.

Individual relative changes (% of baseline values) in weight, BMI, and BMI z-score can be found in Fig. 1. The highest decrease in weight (−8.9 %) and BMI (−13.3 %) was observed in a participant with SOb who attended ≥80 % of all the exercise. Conversely, the highest increase in weight (17.0 %) and BMI (12.9 %) was observed in a participant with SOb with low attendance.

A time-by-group (no-SOb/SOb) effect was found in Central FM and FBFM, with the participants with SOb showing a higher decrease and increase in Central FM and FBFM, respectively (*p* < 0.05) (Table 2).

A time-by-attendance effect was found in weight, BMI, BMI z-score, WHtR, TBFM, BFM, Trunk FM, and MM. Participants who attended at least 80 % of all exercise sessions showed a higher decrease in weight, BMI, BMI z-score, WHtR, TBFM, BFM, and Trunk FM, as well as a higher increase in MM (*p* < 0.05) (Table 2).

### 3.2 Within and between-group changes in clinical parameters

Overall, participants showed over time improvements in cIMT/diameter ratio and VO_2_ peak (*p* < 0.05). In addition, a normalization in high BP (*n* = 10), IR (*n* = 8), Dyslipidemia (*n* = 10), high CRP (*n* = 4), high ALT (*n* = 7), and a remission in metabolic syndrome (*n* = 6) was observed (*p* < 0.05) (Table 3).

**Table 3:** Within and between-group differences in clinical parameters, at baseline and over time, in adolescents with/without severe obesity.

	no-SOb (*n* = 26)		SOb (*n* = 20)		Time	Time∗Group	Time∗Attendance
*Variable*	*Baseline*	6 months	*Baseline*	6 months	*β* (95 % CI)	*β* (95 % CI)	*β* (95 % CI)
cIMT/Diameter ratio	10.34 ± 22.74	92.80 ± 19.62	10.01 ± 22.35	94.98 ± 21.24	**−21.87 (-35.87, -8.17)**	2.30 (−15.41, 20.00)	**−20.10 (-35.95, -4.25)**
VO_2_ peak (ml/kg/min)	21.01 ± 2.51	22.46 ± 2.77	19.56 ± 2.47	19.81 ± 3.32	**1.91 (0.79, 3.03)**	−1.06 (−2.46, 0.33)	**2.84 (1.84, 3.84)**

			Normalization		
	***n* (%)**	***n* (%)**	**no-SOb**	**SOb**	***p***
High BP a	5 (19.2) ∗	11 (55.0) ∗	4 (80.0)	6 (54.5)	0.234
IR a	8 (30.8) ∗	12 (60.0) ∗	1 (12.5)	7 (58.3)	0.**006**
Dyslipidemia a	10 (38.5)	10 (50.0)	6 (60.0)	4 (40.0)	0.802
High CRP a	5 (19.2)	6 (30.0)	3 (60.0)	1 (16.7)	0.435
High ALT a	3 (11.5)	5 (25.0)	3 (100)	4 (80.0)	0.428
Metabolic Syndrome a	3 (11.5)	3 (15.0)	3 (100)	3 (100)	0.730

ALT, alanine aminotransferase; BP, blood pressure; cIMT, carotid intima-media thickness; CRP, C-reactive protein; IR, insulin resistance; SOb, severe obesity (BMI z-score ≥3).

∗ Statistically different at baseline (*p* ≤ 0.05).

Bold β s indicates a significant (*p* ≤ 0.05) time, time-by-group (SOb), and time-by-attendance (≥80 % of all exercise sessions), respectively. Standardized β s adjusted for age, sex, Tanner stage and type of exercise (High-intensity interval training/Traditional training).

a The cutoff values for metabolic variables are presented in Supplemental Table 1.

No significant time-by-group interactions were found in cIMT/diameter ratio nor in VO_2_ peak. Nevertheless, a higher percentage of IR normalization was observed in adolescents with SOb, compared to no SOb (58.3 vs. 12.5 %, *p* = 0.006) (Table 3).

A time-by-attendance effect was found in cIMT/diameter ratio and VO_2_ peak. Participants who attended at least 80 % of all exercise sessions showed a higher decrease and increase in cIMT/diameter ratio and VO_2_ peak, respectively (*p* < 0.05) (Table 3).

### 3.3 Within and between-group changes in physical activity and energy intake

Overall, an over time increase in MPA, VPA, MVPA and PA enjoyment was observed (*p* < 0.05) (Table 4).

**Table 4:** Within and between-group differences in physical activity and energy intake, at baseline and over time, in adolescents with/without severe obesity.

	no-SOb (*n* = 26)		SOb (*n* = 20)		Time	Time∗Group	Time∗Attendance
*Variable*	*Baseline*	6 months	*Baseline*	6 months	*β* (95 % CI)	*β* (95 % CI)	*β* (95 % CI)
Stationary time (min/day)	609.6 ± 128.6	584.9 ± 158.3	574.6 ± 142	571.2 ± 147.8	−11.4 (−99.4, 76.7)	25.1 (−55.7, 105.9)	14.9 (−66.1, 96)
LPA (min/day)	68.0 ± 39.9	73.2 ± 43.5	73.8 ± 54.3	85.2 ± 56.8	0.2 (−0.4, 0.7)	6.2 (−26.7, 39.0)	4.1 (−28.8, 36.9)
MPA (min/day)	35.8 ± 22.8	64.5 ± 39.8	31.9 ± 15.3	61.6 ± 34.2	**0.8 (0.5, 1.1)**	1.1 (−15.7, 17.8)	**26.1 (-11.4, 40.9)**
VPA (min/day)	4.9 ± 4.8	11.5 ± 9.7	5.2 ± 3.5	13.9 ± 8.4	**1.4 (0.9, 1.8)**	2.2 (−2.7, 7.1)	**9.5 (5.5, 13.5)**
MVPA (min/day)	40.8 ± 23.2	76.0 ± 46.6	37.1 ± 17.4	75.6 ± 40.2	**0.9 (0.6, 1.2)**	3.2 (−16.4, 22.9)	**35.6 (19.2, 52.1)**
PA enjoyment	62.4 ± 9.6	68.6 ± 6.3	59.3 ± 8.8	63.1 ± 10.3	**4.8 (1.8, 7.9)**	−2.2 (−6.6, 2.3)	1.9 (−2.6, 6.4)
Energy intake (kcal/day)	1341.4 ± 279.8	1233.2 ± 323.6	1322.7 ± 362.0	1307.6 ± 457.4	−109.1 (−244.3, 26.2)	120.8 (−78.4, 320.0)	**−218.0 (-401.3, -34.7)**

LPA, light physical activity; MPA, moderate physical activity; MVPA, moderate-vigorous physical activity; PA, physical activity; SOb, severe obesity (BMI z-score ≥3); VPA, vigorous physical activity.

Bold β s indicates a significant (*p* ≤ 0.05) time, time-by-group (SOb), and time-by-attendance (≥80 % of all exercise sessions), respectively. Standardized β s adjusted for age, sex, Tanner stage and type of exercise (High-intensity interval training/Traditional training).

No significant time-by-group interactions were found in PA nor energy intake (Table 4).

A time-by-attendance effect was found in MPA, VPA, MVPA, and energy intake. Participants who attended at least 80 % of all exercise sessions showed a higher increase in MPA, VPA, and MVPA, as well as a higher decrease in energy intake (*p* < 0.05) (Table 4).

No differences in attendance were found between SOb and no-SOb participants, with 40 % of participants with SOb and 46.2 % of those with no-SOb attending at least 80 % of all exercise sessions.

### 3.4 Over time differences between adolescents with SOb exposed to exercise-based intervention and those in the control group

Participants with SOb exposed to the exercise-based intervention showed improvements in BMI, WHtR, BFM, SMM, as well as in stationary time and MVPA, compared to an impairment observed in the CG (*p* < 0.05) (Supplemental Table 2).

### 3.5 Adverse effects

Three distinct participants (SOb *n* = 2) reported pain/discomfort in knee and ankle joints on different occasions when performing aerobic exercise on a step platform, despite the adjustment of steps' height to the participants’ height. No other unintended events were reported during or as a consequence of the physical exercise sessions. It should be noted that step platforms were not used thereafter (data not shown).

## 4 Discussion

This study aimed to investigate the efficacy and safety of a high-intensity exercise-based intervention on body composition and cardiometabolic health in adolescents with SOb, compared to those without SOb, since new effective and sustainable treatment options are needed.

According to study results, overall improvements in anthropometrics, body composition, and clinical parameters were found in both participants with SOb and no-SOb in response to physical exercise, with no differences between groups, to the exception of central FM (%/TBFM), FBFM (kg) and IR normalization. These results suggest that adolescents with SOb may have a similar response to exercise to those without SOb. In fact, adolescents with SOb showed higher improvements in central FM and FBFM, which may have led to the observed normalization of IR [25]. In addition, when controlling for baseline values, the highest improvement in weight (−8.9 %) and BMI (−13.3 %) was observed in a participant with SOb (Fig. 1).

Additional analyses showed significant over time differences in BMI, WHtR, BFM, and SMM between adolescents with SOb exposed to the exercise-based intervention compared to those allocated to the CG, highlighting the positive effect of exercise on this specific population.

Interestingly, participants with SOb exposed to the exercise-based intervention tended to have higher improvements in BMI z-score than those in the CG, but this difference was not statistically significant. This fact may be partly explained by the small number of participants in each group and by the attendance at the exercise sessions. Although no differences in attendance were found between participants with and without SOb, more than half of the participants (60.0 % with SOb and 53.8 % without SOb) attended less than 80 % of all exercise sessions, which may have mitigated the potential effect of physical exercise, also increasing interindividual variability, as can be observed in Fig. 1. In fact, a time-by-attendance interaction was found in most anthropometric and body composition variables and clinical parameters, supporting the relevance of exercise volume and frequency on energy expenditure and health-related outcomes [18,26].

The low attendance observed in present study aligns with findings from other exercise-based interventions [27], highlighting the importance of identifying factors that may influence attendance in this population. For instance, concerns related to body image and psychological states may reduce attendance and overall adherence to exercise. These psychological barriers suggest a need for integrated support strategies to enhance engagement and maximize the benefits of exercise interventions.

Participants with SOb exposed to the exercise intervention showed a mean decrease in BMI z-score and BMI of −0.16 (95 %CI: 0.25, −0.07) and −0.51 (95 %CI: 1,20 0.17), respectively (Table 2). Although these results may have led to improvements in cardiometabolic health [19], they are far below those expected from bariatric surgery. At 6 months, a mean reduction of 11.6 kg/m^2^, 14.1 kg/m^2,^ and 16.6 kg/m^2^ following adjustable gastric band, sleeve gastrectomy, and Roux-en-Y gastric bypass have been reported, respectively [28]. However, compared to GLP1-RA (namely Liraglutide, that has shown higher efficacy in weight loss, compared to other drugs) [29] the exercise-based intervention suggests overlapping results on BMI z-score (−0.16 vs. −0.14) [30]. Although limited evidence exists in adolescents, the combination of the two therapeutics (i.e., GLP-1 receptor agonists and high-intensity exercise) may further enhance obesity-related outcomes [31], with exercise playing a key role in maintaining weight loss after the discontinuation of pharmacological treatment [32], as it has been suggested in adults.

Similarly to other therapeutic options, the exercise-based intervention depended on compliance, with more marked benefits, including daily physical activity and total energy intake, in those with higher attendance. Along with attendance, the maintenance of exercise levels would lead to higher benefits and improvements in the long run. Yet, conversely, to other therapeutics, no adverse or unintended effects were reported.

## 5 Limitations

The low number of participants included is the main limitation of the present study, as it increases Type II errors. Another possible limitation may be that EGII was exposed to two distinct physical exercise protocols. Half of the included participants performed a traditional training protocol, and the other half combined high-intensity interval training [18]. Although this fact may have biased study results, it should be noted that data analyses were adjusted to the type of exercise. Moreover, attendance, but not the exercise protocol itself, was found to be the best predictor of the observed improvements, and according to previous studies, no differences may exist between protocols regarding most of the health-related outcomes, such as IR normalization [33]. Missing DXA data in the CG, which did not allow a broader comparison between participants with SOb from EGII and CG, may also be considered a limitation. However, bioelectrical impedance has shown to be a reliable method to assess body composition [34].

## 6 Conclusion

Although efforts to improve attendance must be made, this study shows that high-intensity physical exercise-based interventions should be considered a viable obesity treatment option in adolescents with SOb. More intensive exercise-based interventions, including higher volume and frequency, may further improve health-related outcomes in this population and, therefore, could be considered a first-line therapeutic option alongside other lifestyle changes. The combination of high-intensity physical exercise-based interventions with GLP1-RA medications may further improve the outcomes, thus it should be considered as a second-line therapeutic option. Yet, more evidence is needed on that combination.

## Author contributions

Conception and design of the study: AVS, LBS, and HF. Development of the analysis, the acquisition of the data, and performing the data analysis: AVS. Principal writer of the article: AVS. Drafting of the article and revising it critically: AVS, FF, LBS, and HF. Approval of the final version to be submitted: All authors.

## Ethics

The trial was approved by the Faculty of Medicine Ethics Committee (University of Lisbon, 271/2016), following the 1964 Helsinki Declaration and its later amendments or comparable ethical standards. Informed assent/consent was signed by all the participants and caregivers.

## Artificial intelligence (AI)

During the preparation of this work the authors did not use AI assisted technologies.

## Declaration of competing interests

The authors declare no competing financial interests or personal relationships that could have influenced the work reported in this manuscript.
