# Supplementary material for: Effect and safety of a physical exercise-based intervention on body composition and cardiometabolic health of adolescents with severe obesity. Secondary analysis from the PAC-MAnO trial
Source: Obes Pillars. 2025 Jun 26;16:100190. doi: 10.1016/j.obpill.2025.100190 (PMC12275681; doi:10.1016/j.obpill.2025.100190)
Supplement: Multimedia component 1 [file mmc1.docx]

**Supplemental Table 1.** Cutoff values used to determine the presence of metabolic obesity-related comorbidities.

ALT, alanine aminotransferase; CRP, C-reactive protein; DBP, diastolic blood pressure; HDL-C, high-density lipoprotein cholesterol; HOMA-IR, insulin resistance homeostasis model assessment; LDL-C, low-density lipoprotein cholesterol; SBP, systolic blood pressure; TC, total cholesterol; TG, triglyceride.

| ***Outcome*** |  | **CG (*n*=16)** | | | **EG (*n*=20)** | |  |
| --- | --- | --- | --- | --- | --- | --- | --- |
|  |  | ***n* (%)** | | | ***n* (%)** | | ***p*** |
| Ethnicity (Caucasian) |  | 14 (87.5) | |  | 13 (65.0) |  | .202 ^a^ |
| Sex (Girls) |  | 11 (68.8) | |  | 6 (30.0) |  | **.021** ^a^ |
| Tanner stage | II | 3 (18.8) | |  | 3 (15.0) |  | .796 ^a^ |
|  | III | 0 (0.0) | |  | 1 (5.0) |  |  |
|  | IV | 4 (25.0) | |  | 6 (30.0) |  |  |
|  | V | 9 (56.3) | |  | 10 (50.0) |  |  |
|  |  | ***Baseline*** | | ***6 months*** | ***Baseline*** | ***6 months*** |  |
|  |  | **Mean ± SD** | | | **Mean ± SD** | | ***p* ^(1)^** |
| Age (years) |  | 13.9 ± 1.8 |  | | 14.2 ± 2.1 |  |  |
| Weight (kg) |  | 92.4 (13.6) * | | 97.1 (10.9) | 102.4 (20.8) * | 106.5 (22.6) | .102 ^b^ |
| BMI (kg/m^2^) |  | 36.63 (4.49) * | | 38.47 (3.96) | 37.32 (3.50) * | 37.24 (6.03) | **.043 ^b^** |
| BMI z-score |  | 3.49 (0.66) * | | 3.48 (0.63) | 3.50 (0.55) * | 3.36 (1.17) | .149 ^b^ |
| WHtR |  | 0.69 ± 0.07 | | 0.71 ± 0.05 | 0.71 ± 0.06 | 0.70 ± 0.07 | **.026 ^c^** |
| BFM (%) ^(2)^ |  | 47.4 ± 4.5 | | 48.6 ± 4.1 | 47.0 ± 4.8 | 45.0 ± 6.3 | **.018 ^c^** |
| MM (%) ^(2)^ |  | 29.2 ± 2.8 | | 28.4 ± 2.9 | 29.7 ± 3.0 | 31.0 ± 3.9 | **.013 ^c^** |
| Stationary time (min/day) |  | 603.4 (96.1) | | 682.9 (103.4) | 608.6 (196.3) | 592.2 (165.2) | **.007 ^b^** |
| LPA (min/day) |  | 50.6 (83.1) | | 53.7 (49.4) | 50.4 (80.4) | 62.4 (52.4) | .158 ^b^ |
| MVPA (min/day) |  | 25.9 (12.2) | | 27.6 (18.0) | 35.6 (22.5) | 74.1 (59.0) | **<.001 ^b^** |
| Energy intake (kcal/day) |  | 1050.4 ± 281.8 | | 1199.8 ± 219.0 | 1322.7 ± 362.0 | 1307.6 ± 457.4 | .345 ^c^ |

BFM, body fat mass; BMI, body mass index; LPA, light physical activity; MM, muscle mass; MVPA, moderate-vigorous physical activity; WHtR, waist-height ratio.

^(1)^ Over time between-group differences; ^(2)^ Body composition data assessed by bioelectrical impedance.

^a, b, c^ Between-group differences analyzed with Chi-squared, Mann-Whitney U test, and Independent-sample *t*-test, respectively. For non-normal distributed variables Median (Interquartile Range) is presented.

* Statistically different at baseline (*p* ≤.05).

**Supplemental Table 2.** Over time differences in adolescents with severe obesity allocated to the exercise-based intervention (Experimental Group, EG) compared to controls (CG).
